# Age-dependent expression and antiviral activity of interferon epsilon in respiratory epithelium

**DOI:** 10.1128/jvi.00578-25

**Published:** 2026-05-12

**Authors:** Mary McCabe, Helen E. Groves, Erin Getty, Emma Campbell, Connor G. G. Bamford, David G. Courtney, Guillermo Lopez Campos, Michael D. Shields, Ultan F. Power

**Affiliations:** 1 Wellcome-Wolfson Institute for Experimental Medicine, Queen's University Belfast 231327, Belfast, United Kingdom; 2 School of Biological Sciences & Institute for Global Food Security, Queen's University Belfast 98871, Belfast, United Kingdom; University Medical Center Freiburg, Freiburg, Germany

**Keywords:** respiratory syncytial virus, innate immunity, interferons, interferon epsilon, sendai virus, SARS-CoV-2

## Abstract

Respiratory syncytial virus (RSV) disease burden is greatest between 6 weeks and 6 months of life, with young age the most common risk factor among hospitalized children. A robust innate immune response in the airway epithelium is crucial for mitigating RSV-associated disease, but early-life immune responses to infection remain largely unexplored. RNA-seq analysis of RSV-infected primary nasal epithelial cell cultures from healthy infants at birth and 1 year revealed diminished expression of interferon epsilon (*IFNE*), a poorly characterized type I IFN, in newborns vs 1 year. We hypothesized, therefore, that *IFNE* plays an important role during infant RSV infection. We found that *IFNE* is basally expressed in airway epithelial cell lines. Recombinant human IFNε (rhIFNε) induced an antiviral state against an RSV clinical isolate and related Sendai virus, but not SARS-CoV-2 under the conditions tested. The antiviral potency of rhIFNε was diminished relative to rhIFN beta (rhIFNβ1) or rhIFN lambda-1 (rhIFNλ1), as evidenced by IC_50_ data. Importantly, rhIFNε induced similar ISGs as rhIFNβ1 but demonstrated a transient temporal expression profile that differed from both rhIFNβ1 and rhIFNλ1. These results suggest that lower IFNε expression at birth may contribute to increased susceptibility to severe RSV-associated disease, offering insights into potential interventions.

**IMPORTANCE:**

Respiratory syncytial virus (RSV) is a major pediatric respiratory pathogen. Despite advances in vaccine development and monoclonal antibody prophylaxis, the immune factors driving RSV-associated disease remain unclear. Notably, severe disease is not limited to recognized high-risk groups; most affected children share only young age as a risk factor. To better understand virus-host interactions in early life, we previously derived primary airway epithelial cell cultures from the same healthy newborns at birth and at 1 year, infected them with a clinical RSV isolate, and performed total RNA sequencing. In this study, we present evidence that the transcriptional expression of interferon epsilon (*IFNE*), a relatively understudied type I interferon, varies with chronological age, coinciding with the period of the greatest burden of severe RSV-associated disease. We also show that recombinant human IFNε exhibits antiviral activity against RSV in airway epithelial cells, although with reduced potency compared to other type I and III interferons. These results expand our understanding of interferon responses during early-life RSV infection and suggest that reduced IFNε expression in newborns may partly explain their increased vulnerability to severe disease.

## INTRODUCTION

Respiratory syncytial virus (RSV), a single-stranded negative-sense RNA virus of the *Pneumoviridae* family and *Orthopneumovirus* genus, is the commonest cause of acute lower respiratory tract infection (ALRTI) in infants globally (1). Annually, ~33 million RSV LRTIs occur in children <5 years old, resulting in ~101,400 infant deaths (1). Despite recent pharmacological advancements (2–6), including the introduction of a maternal vaccine that provides passive immunity via transplacental antibody transfer, and the development of an improved monoclonal antibody administered to infants considered at high risk of severe disease, there are still no antivirals or vaccines approved for direct administration to children under 2 years old, the age group in which the disease burden is greatest (1, 7). While epidemiological data have identified risk factors (8–14), most infants hospitalized share only the risk factor of age, suggesting host intrinsic features predispose them to severe disease. Because of the current incomplete understanding of RSV-host interactions in early life, we cannot predict which infants will experience severe disease and, thereby, prioritize prophylactic treatment. As RSV initially targets the ciliated cells of the nasopharynx (15–17), efficient innate immune responses play a vital role in determining RSV infection outcomes. Yet, whether and how these responses develop over time, and why infants <6 months are more frequently susceptible to severe disease, is unknown.

Understanding the innate immune response to infection is important in early life when traditionally infant adaptive immune responses are considered limited by antigenic naivety (18). Robust and timely innate immune responses are key to shaping effective adaptive immunity, and therefore, enhancing localized mucosal responses may help support still-developing infant systemic immunity. Importantly, natural infection with RSV does not confer lifelong immunity (19), and therefore, the innate immune system plays a vital role in bridging the gap when antibody-mediated protection, whether naturally acquired or passively transferred, is insufficient. A key challenge is that most infants with severe RSV-associated disease do not present with identifiable high-risk factors and, therefore, may not meet the criteria for current prophylactic interventions. There are also significant disparities in access to vaccines and monoclonal antibodies, particularly in low- and middle-income countries where the burden of severe RSV-associated disease is greatest (1). By harnessing or enhancing existing localized mucosal innate immune responses to viral infection, better protection for these broader populations could possibly be provided. Exploring understudied components of the innate immune response could also lead to the identification of biomarkers and pathways that support risk stratification and guide the development of targeted interventions for populations currently lacking effective treatment options. An approach focused on host innate immunity may address unmet needs in populations traditionally considered relatively low-risk but still vulnerable, while also offering potential treatment options for individuals with underdeveloped or waning systemic immunity who are at high risk for severe RSV-associated disease.

Interferons (IFN) are a crucial innate barrier against viral infection and dissemination (20, 21). In the respiratory epithelium, type I (IFNα and -β) and type III (IFNλ1-λ3) IFNs play significant roles. Recently, Taveras et al. determined that all IFN types (I, II, and III) from nasal samples were significantly increased in children (>6 years) vs infants (<6 months) and higher in milder outpatient RSV infections compared to severe inpatient infections (22). Furthermore, we observed an increase in mean IFNλ1 protein secretion at 1 year vs birth within paired birth and 1-year primary nasal cell samples (23). These results suggest that strong IFN responses are a critical component of determining disease severity. As such, more robust IFN responses to RSV infection with increasing chronological age may contribute to protection against severe disease in older infants and adults.

IFN epsilon (*IFNE*/IFNε) is a highly conserved type I IFN recognized for its multifaceted antimicrobial properties in the female reproductive tract (FRT) and its expression at other mucosal interfaces, including the lungs (24–27). In contrast to other type I IFN subtypes, IFNε is 100- to 1,000-fold less potent and is expressed at high levels basally (28–30). Interestingly, Thwaites et al. described a trend for increased expression of type I IFNs (*IFNA1*, *IFNB1,* and *IFNE*) in infants with moderate RSV ALRTI relative to severe cases (31), and *IFNE* was upregulated in children and young adults with severe acute respiratory syndrome coronavirus 2 (SARS-CoV-2) infection (32). Furthermore, *IFNE* was observed to be differentially expressed in children with long-coronavirus disease (COVID) symptoms, with lower expression of *IFNE* in those with long-COVID compared to healthy controls (33). Therefore, despite its conserved mucosal antiviral activity, understanding of the local expression and function of IFNε in airway epithelium against respiratory viruses, and its possible contributions to viral disease severity, remains limited.

In this study, we report for the first time an increase in *IFNE* expression over the first year of life, beginning with low levels at birth and rising to significantly higher levels by 1 year of age. We confirmed its basal expression in immortalized airway epithelial cells and assessed its antiviral activity against a clinical isolate of RSV and a related RNA virus, Sendai virus, and SARS-CoV-2. We determined the half-maximal inhibitory concentration (IC_50_) of rhIFNε against RSV compared to other relevant mucosal IFNs and characterized the induction of downstream interferon-stimulated genes (ISGs). These results suggest an underappreciated role of IFNε in the induction of protective antiviral responses in the respiratory epithelium.

## MATERIALS AND METHODS

### Cell culture

BEAS-2B cells (kindly provided by Cliff Taggart, Queen’s University Belfast) were maintained in 1 g/L (low) glucose DMEM with 10 units/mL Pen/Strep and 5% FBS. HEp-2 cells (kindly provided by Prof. Ralph Tripp, University of Georgia) were maintained in 4.5 g/L (high) glucose DMEM with 10 units/mL Pen/Strep and 5% FBS. A549, Vero E6, and Vero expressing ACE2 and TMPRSS2 (VAT) cells were maintained in 4.5 g/L (high) glucose DMEM with 10 units/mL Pen/Strep and 10% FBS. A549 expressing human ACE2 and TMPRSS2 (AAT) cells were maintained in 4.5 g/L (high) glucose DMEM with 10 units/mL Pen/Strep, 10% FBS and maintained under antibiotic selection with Hygromycin B (25 µg/mL) and Geneticin (25 µg/mL).

Nasal brushings were performed on healthy newborn term infants (37–42 weeks gestation) and preterm infants (28–34 weeks gestation) at birth and repeated in the same infants at 1-year old, as previously described (34). Harvested pediatric nasal cells were cultured to differentiation into well-differentiated primary nasal epithelial cell (WD-PNEC) cultures using established methods (16, 35) and infected with a multiplicity of infection (MOI) 3 in duplicate with a clinical isolate of RSV, designated BT2a, or mock-infected as previously described (36).

### RNA-Seq

Total RNA was extracted from WD-PNEC cultures at 96 hours post-infection (hpi) (High pure RNA isolation kit, Roche diagnostics, Indianapolis, USA), and RNA quality was determined (Agilent RNA 6000 Nano Kit) using the 2100 Bioanalyzer Instrument (Agilent Technologies). Total RNA sequencing was conducted (23), and the data have been deposited in the NCBI GEO database under accession number GSE231666.

### Virus stocks and titrations

RSV BT2a (clinical isolate) was isolated and characterized as previously described (36). RSV A2/mKate2 (a recombinant RSV reporter virus expressing Katushka, a far-red fluorescent protein) was rescued from an infectious clone and helper plasmids kindly provided by Dr Martin Moore (Emory University, Atlanta, GA, USA). Sendai virus/eGFP (rSeV/eGFP) was generated by reverse genetics (37). SARS-CoV-2 Delta Variant was isolated from nasal swabs (38). EMCV-Rueckert was obtained as described previously (39). Viral infections were performed at the MOI stated in each figure legend by adding diluted virus suspensions to the apical compartment of WD-PNECs or directly onto cell lines in serum-free medium. Cells were then infected in serum-free DMEM. Following infection, cells were incubated for 2 h at 37°C. After 2 h, the viral inoculum was removed and washed three times with serum-free DMEM to remove unattached virus; low-serum DMEM was replaced and incubated at 37°C. HEp-2 cells were used to titrate RSV BT2a, and Vero E6 cells were used to titrate EMCV by tissue culture infectious dose 50 (TCID_50_) assays. SARS-CoV-2 plaque assays were performed on VAT cells (38).

### RNA extraction and RT-qPCR

RNA was extracted according to the manufacturer’s instructions (High pure RNA isolation kit, Roche), and RNA concentration and purity were determined (Nanodrop One, Thermo Fisher Scientific). cDNA synthesis was performed in a 96-well thermocycler (Bio-Rad) using a High-Capacity cDNA Reverse Transcription Kit (Applied Biosystems) according to the manufacturer’s instructions. For the target genes, *IFNE* (assay ID Hs00703565_s1)*, IFNL1* (assay ID Hs00601677_g1), *IFNB1* (assay ID Hs01077958_s1)*, MX1* (assay ID Hs00895608_m1)*, ISG15* (assay ID Hs01921425_s1)*, IFIT1* (assay ID Hs03027069_s1)*, RSAD2* (assay ID Hs00369813_m1)*, IRF1* (assay ID Hs00971965_m1), *CXCL10* (assay ID Hs00171042_m1), YWHAZ (assay ID Hs01122445_g1), TBP (assay ID Hs00427620_m1), and IPO8 (assay ID Hs00914057_m1) Thermo Fisher Scientific TaqMan ready-to-use primer/probes were purchased and employed with the LightCycler 480 Probes Master Mix (Roche). Relative quantification analysis of gene expression was performed using LightCycler 96 software (Roche). The Relative Quantification Cycle (Cq) of the sample was determined by comparing the target gene to three reference housekeeping genes: *YWHAZ*, *TBP*, and *IPO8*.

### Recombinant human protein

Cell lines were pretreated with recombinant human interferon epsilon (rhIFNε) (R&D Systems, Minneapolis, MN, USA), rhIFN beta (rhIFNβ1) (PeproTech), and rhIFN lambda-1 (rhIFNλ1) (PeproTech) in low-serum DMEM for intervals stated within each figure legend.

### Cell imaging and analysis

The mean percent fluorescence ratio of RSV A2/mKate2 and rSeV/eGFP infected cells was measured using the brightfield and red/green fluorescence channels of the Celigo Imaging Cytometer (Nexcelom). All data are presented as percent fluorescence ratio [(red or green confluence/brightfield confluence) × 100].

### Generation of BEAS-2B *IFNLR1*^*−/−*^ cells

BEAS-2B cells overexpressing Cas9 were generated via lentiviral transduction of a third-generation lentiviral Cas9 expression plasmid (Addgene #52962), blasticidin selection (Gibco, 10 µg/mL), and single-cell clonal isolation. Western blotting was used to confirm the stable expression of Cas9 (Sigma-Aldrich, F3165). To generate *IFNLR1* BEAS-2B clonal cell line knockouts, a single-guide RNA (sgRNA) (priCRISPR.IFNLR1.1(+) CACCGGTATTCGGACTCCACCCAG) was designed. For the sgRNA, a forward and reverse oligonucleotide sequence was synthesized (Eurofins), annealed, and inserted into the lentiGuide-Puro vector via BsmB1-v2 (NEB) restriction digestion and T4 DNA ligase (NEB) mediated ligation. Vector-sgRNA DNA was then transformed into DH10B competent cells (Thermo Fisher Scientific) and spread onto Ampicillin selection plates (50 μg/mL). Single colonies were picked and miniprepped using PureLink HiPure Plasmid Miniprep kit according to manufacturer’s instructions (Invitrogen). BEAS-2B *IFNLR1* knockout cell lines were generated via lentiviral transduction of a third-generation lentiGuide-Puro plasmid (Addgene #84752) with inserted sgRNA sequence, puromycin (Gibco) selection, and single-cell clonal isolation.

### Statistical analysis

Statistical analyses included an unpaired two-tailed Student’s *t*-test and one-way analysis of variance (ANOVA) to assess the significance of test conditions, as stated in the figure legends. For the time course profiles, we calculated the Area under Curve (AUC) and then used this summary measure for comparisons using one-way ANOVA. Statistical analysis was set at *P < 0.05, **P < 0.01, ***P < 0.001, ****P < 0.0001. Data presented as means ± standard error of the means (SEM). Data were analyzed using GraphPad Prism 10 (GraphPad Software, Inc, La Jolla, CA).

## RESULTS

### Basal expression of *IFNE* increases during the first year of life

**Fig 1:**
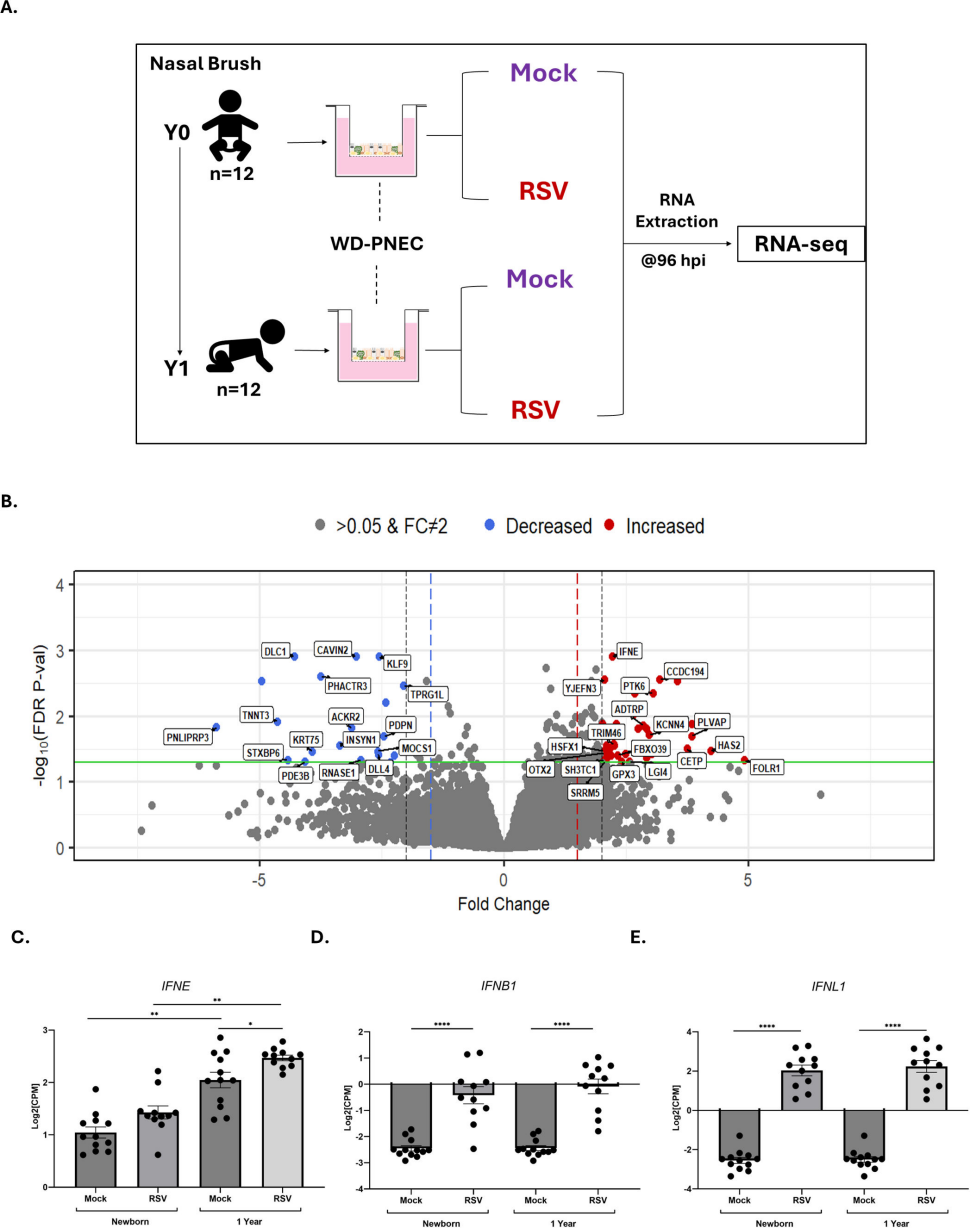
Increase in *IFNE* expression during the first year of life. (**A**) Summary of RNA-Seq experimental set-up. WD-PNEC cultures (*n* = 12 pediatric donors) were infected with clinical isolate RSV BT2a (MOI = 3) or mock-infected. RNA was extracted at 96 hpi, and total RNA sequencing was conducted. (**B**) Volcano plot displaying differentially expressed genes associated with RSV-infected cultures from 1-year-old and newborn donors, with protein-coding genes with an expression log2 fold change (FC) ≥ 2 and log2 FC ≤ −2 highlighted. The *x*-axis depicts the log2 FC differences in gene expression for each comparison of interest, with positive values (in red) displaying significantly increased gene expression (log2 FC ≥ 2) and negative values (in blue) displaying significantly decreased gene expression (log2 FC ≤ −2). Blue and red dashed lines indicate custom log2 FC thresholds at −1.5 (blue) and +1.5 (red), while grey vertical lines indicate the standard fold-change cutoff at ±2 log2 FC**.** The *y-*axis depicts adjusted *P* values (−log10 scale) for each gene (higher *P* values representing greater statistical significance) after adjusting for batch by the year RNA-sequencing was completed. Green horizontal line indicates 0.05 *P*-value significance cut-off. (**C**) *IFNE,* (**D**) *IFNB1,* and (**E**) *IFNL1* log2 counts per million (CPM). Statistical analyses were corrected for multiple testing by applying the Benjamini-Hochberg (BH) *P*-value correction as part of RNA-seq analysis pipeline (*P < 0.05, **P < 0.01, ***P < 0.001, ****P < 0.0001).

In a recent study, we evaluated the impact of gestational and chronological age on RSV-induced airway epithelium innate immune responses using RNA-Seq (23) (Fig. 1A). RNA-seq analysis of RSV-infected WD-PNECs derived from infants sampled at birth and 1-year old revealed 63 differentially expressed genes (DEGs) (42 upregulated, 21 downregulated) with Log2Fold >|2| (Fig. 1B). Included in the 42 upregulated DEGs, *IFNE,* a poorly characterized type I IFN, was significantly increased following RSV infection vs uninfected controls in 1-year children but not newborn infant WD-PNECs (Fig. 1C). Furthermore, *IFNE* expression was higher in 1 year- vs newborn-derived WD-PNECs, both at baseline and following RSV infection (Fig. 1C). The basal expression pattern of *IFNE* is clearly displayed when compared to the expression pattern of *IFNB1* (Fig. 1D) and *IFNL1* (Fig. 1E). These results suggested that *IFNE* may play an unappreciated role in respiratory airway epithelium and contribute to more robust antiviral activities at 1 year compared to newborns.

### Basal expression of *IFNE* in immortalized airway epithelial cells

**Fig 2:**
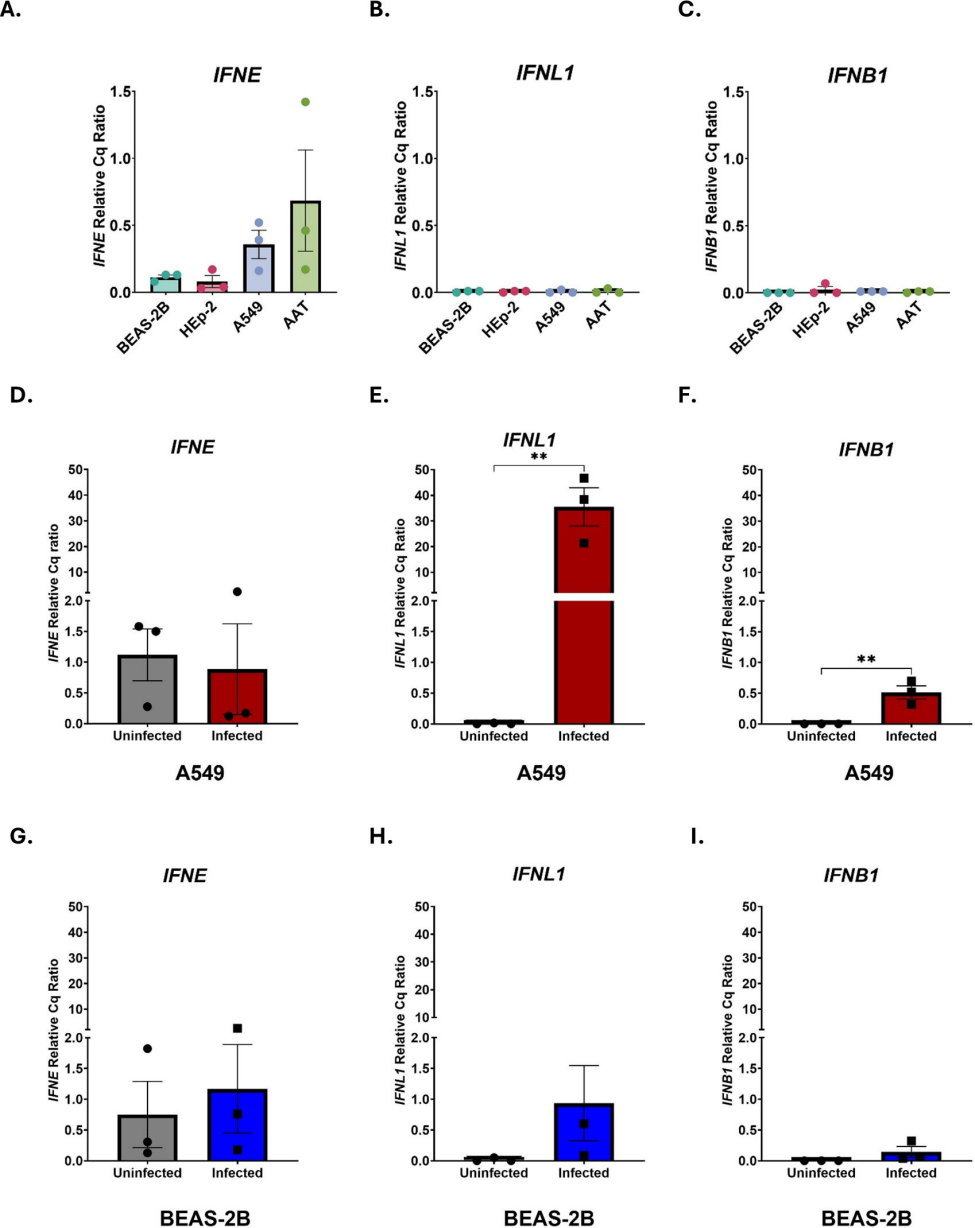
*IFNE* is basally expressed in immortalized airway epithelial cells. BEAS-2B, A549, AAT, and HEp-2 cells were seeded in 24 well plates. Twenty-four hours post seeding total RNA was extracted, reverse transcribed, and subjected to qPCR analysis for *IFNE*, *IFNL1,* and *IFNB1* expression (ROCHE, Lightcycler 480). Graphs show relative ratio values for (**A**) *IFNE*, (**B**) *IFNL1,* and (**C**) *IFNB1* normalized using *YWHAZ*, *IPO8,* and *TBP* as housekeeping genes. Data are presented as mean of triplicate technical replicates (±SEM) for *n* = 3 independent experiments. (**D–F**) A549 or (**G–I**) BEAS-2B cells were mock-infected or infected with RSV-A2/mKate2 (MOI 1). At 48 hpi, total RNA was extracted, reverse transcribed, and subjected to qPCR analysis of *IFNE*, *IFNL1,* and *IFNB1* expression (ROCHE, Lightcycler 480). Graphs show relative ratio values for *IFNE*, *IFNL1,* and *IFNB1* normalized using *YWHAZ*, *IPO8,* and *TBP* as housekeeping genes. Data are presented as the mean of duplicate technical replicates (±SEM) for *n* = 3 independent experiments at 48 hpi. Statistical analyses were conducted using an unpaired two-tailed Student’s *t*-test (*P < 0.05, **P < 0.01).

Next, we assessed the expression of *IFNE* and two other mucosal IFNs within airway epithelial cells, including a type I (*IFNB1*) and a type III (*IFNL1) IFN. IFNB1* was selected as a comparator because it was the only other type I IFN, other than *IFNE*, found to be differentially expressed with infection in the RNA-seq data set (23). *IFNλ1* was chosen as a representative type III IFN as our group showed increased IFNλ1 release from primary cultures derived from infant respiratory tract samples following RSV infection (40). Within common airway epithelial cell lines used to model RSV infection, *IFNE* was basally expressed at low levels, with the highest expression in adenocarcinoma human alveolar basal cells (A549) and A549 cells overexpressing human ACE2 and TMPRSS2 (AAT) (Fig. 2A). Low levels of expression were evident in immortalized human bronchial epithelial cells (BEAS-2B) and human laryngeal carcinoma cells (HEp-2) (Fig. 2A). This contrasted with *IFNL1* and *IFNB1*, which exhibited negligible expression under unstimulated sterile cell-culture conditions (Fig. 2B and C). To further characterize *IFNE* expression, we infected A549 (Fig. 2D through F) and BEAS-2B (Fig. 2G through I) cells with a recombinant RSV reporter virus expressing a far-red fluorescent protein (RSV-A2/mKate2) or mock-infected them. At 48 hpi, *IFNE*, *IFNB1,* and *IFNL1* mRNA expression was higher within A549 cells (Fig. 2D through F) compared to BEAS-2B cells (Fig. 2G through I). The distinct, infection-independent expression pattern of *IFNE* (Fig. 2D and G) becomes particularly apparent when contrasted with the RSV-induced upregulation of *IFNL1* (Fig. 2E and H) and *IFNB1* (Fig. 2F and I).

### IFNε has antiviral activity against RSV that is concentration and cell type dependent

**Fig 3:**
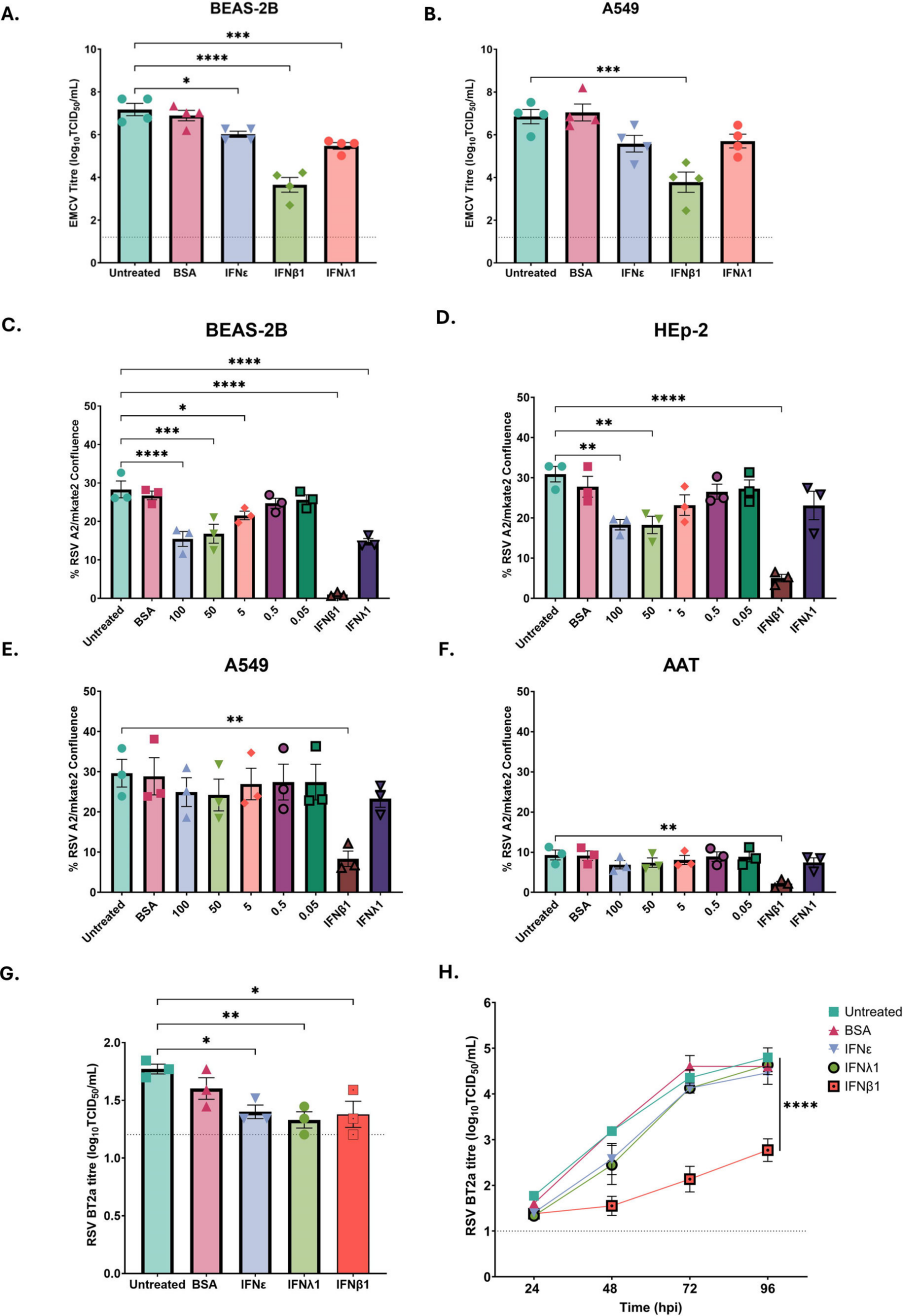
IFNε pretreatment reduces EMCV and RSV replication in BEAS-2B cells. (**A**) BEAS-2B and (**B**) A549 cells were pre-treated for 16 h with an rhIFN (100 ng/mL). Cells were then infected with EMCV (MOI = 1) for 2 h at 37°C. Data are presented as mean of duplicate technical replicates (±SEM) for *n* = 4 independent experiments. Statistical analysis was conducted using one-way ANOVA. (**C**) BEAS-2B, (**D**) HEp-2 (**E**) A549, and (**F**) AAT cells were pre-treated for 16 h with rhIFNε (100, 50, 5, 0.5, and 0.05 ng/mL), rhIFNβ1 or rhIFNλ1 (100 ng/mL). Cells were then infected with RSV-A2/mKate2 (MOI = 0.3). (**C–F**) At 24 hpi, mean % fluorescence ratio was measured using the brightfield and red fluorescence channels of the Celigo Imaging Cytometer (Nexcelom). Data presented as % fluorescence ratio for mean of triplicate technical replicates (±SEM) *n* = 3 independent experiments. Statistical analysis (**C–F**) was conducted using one-way ANOVA. (**G and H**) BEAS-2B cells were pre-treated for 16 h with 100 ng/mL of rhIFN. Cells were then infected with RSV BT2a (MOI = 0.3). Supernatants were collected at 24, 48, 72, and 96 hpi, and TCID_50_ assays were conducted to determine RSV titers. Data are presented as the mean of duplicate technical replicates (±SEM) log_10_ TCID_50_/mL at (**G**) 24 hpi and a (**H**) 24–96 hpi time course for *n* = 3 independent experiments. Statistical analysis for (**G**) was conducted using one-way ANOVA followed by Dunnett's multiple comparisons test and for (**H**) using one-way ANOVA of AUC with Dunnett's multiple comparisons test (*P < 0.05, **P < 0.01, ***P < 0.001, ****P < 0.0001).

To assess if recombinant human IFNε (rhIFNε) protein can induce an antiviral effect within airway epithelial cell lines, BEAS-2B (Fig. 3A) and A549 (Fig. 3B) cells were pre-treated with rhIFNε, rhIFNβ1, and rhIFNλ1 protein (100 ng/mL) and infected with the highly IFN-sensitive virus encephalomyocarditis virus (EMCV-Rueckert) (MOI 1). rhIFNε pretreatment resulted in a 1.15-log_10_ reduction in EMCV titer vs the untreated in BEAS-2B cells (Fig. 3A). This was similar to rhIFNλ1 pretreatment (1.7-log reduction) but much less effective than rhIFNβ1 pretreatment (3.5-log_10_ reduction). There was a non-statistically significant trend toward a reduction in virus titers within A549 cells (Fig. 3B).

To explore the antiviral activity of rhIFNε against RSV, BEAS-2B (Fig. 3C), HEp-2 (Fig. 3D), A549 (Fig. 3E), and AAT (Fig. 3F) cells were pretreated with rhIFNε at a range of doses (100, 50, 5, 0.5, and 0.05 ng/mL) and infected with RSV-A2/mKate2 (MOI = 0.3). A range was selected, as prior to this study, there was no evidence of IFNε’s antiviral effect against respiratory viruses. At 24 hpi, the mean % fluorescence ratio was measured using the brightfield and red fluorescence channels of the Celigo Imaging Cytometer (Nexcelom). BSA pretreatment (5 µg/mL) was a negative control for the recombinant protein stabilization carrier, and rhIFNβ1 and rhIFNλ1 pretreatment (100 ng/mL) were used as positive controls to ensure cell responsiveness to IFNs. Using fluorescence as a surrogate marker of viral spread, rhIFNε pretreatment resulted in a significant reduction in virus fluorescence in BEAS-2B (Fig. 3C) and HEp-2 cells (Fig. 3D), but not in A549 or AAT cells (Fig. 3E and F), although there was a trend toward reduction in the latter cell lines. Reduction in viral spread was dose-dependent, with 100 ng/mL of rhIFNε resulting in the greatest reduction compared to untreated controls (1.8-fold reduction) in the most responsive cell line (BEAS-2B) (Fig. 3C). This was less potent than rhIFNβ1 (27-fold reduction) but similar to rhIFNλ1 (1.9-fold reduction) (Fig. 3C). RSV-A2/mKate2 spread was much lower in AAT cells (Fig. 3F) compared to their parental counterpart, A549 cells. To assess the antiviral effect against RSV BT2a (36), BEAS-2B cells were pre-treated with 100 ng/mL of rhIFNε, rhIFNβ1, and rhIFNλ1 and infected with RSV BT2a (MOI 0.3). BEAS-2B cells were selected as they are a non-tumor transformed cell line and more responsive to IFN stimulation. We found a 0.37-Log_10_ reduction at 24 hpi (Fig. 3G) in RSV titers in rhIFNε pre-treated cells compared to untreated controls. A similar reduction was seen with rhIFNβ1 (0.39-Log_10_ reduction) and rhIFNλ1 (0.44-Log_10_ reduction) pre-treatment at 24 hpi. Thereafter, while mean virus growth kinetics were delayed slightly at 48 hpi, titers at 72 and 96 hpi were indistinguishable from untreated or BSA controls (Fig. 3H).

### Relative antiviral activity of IFNε, IFNλ1, and IFNβ1 against RSV

**Fig 4:**
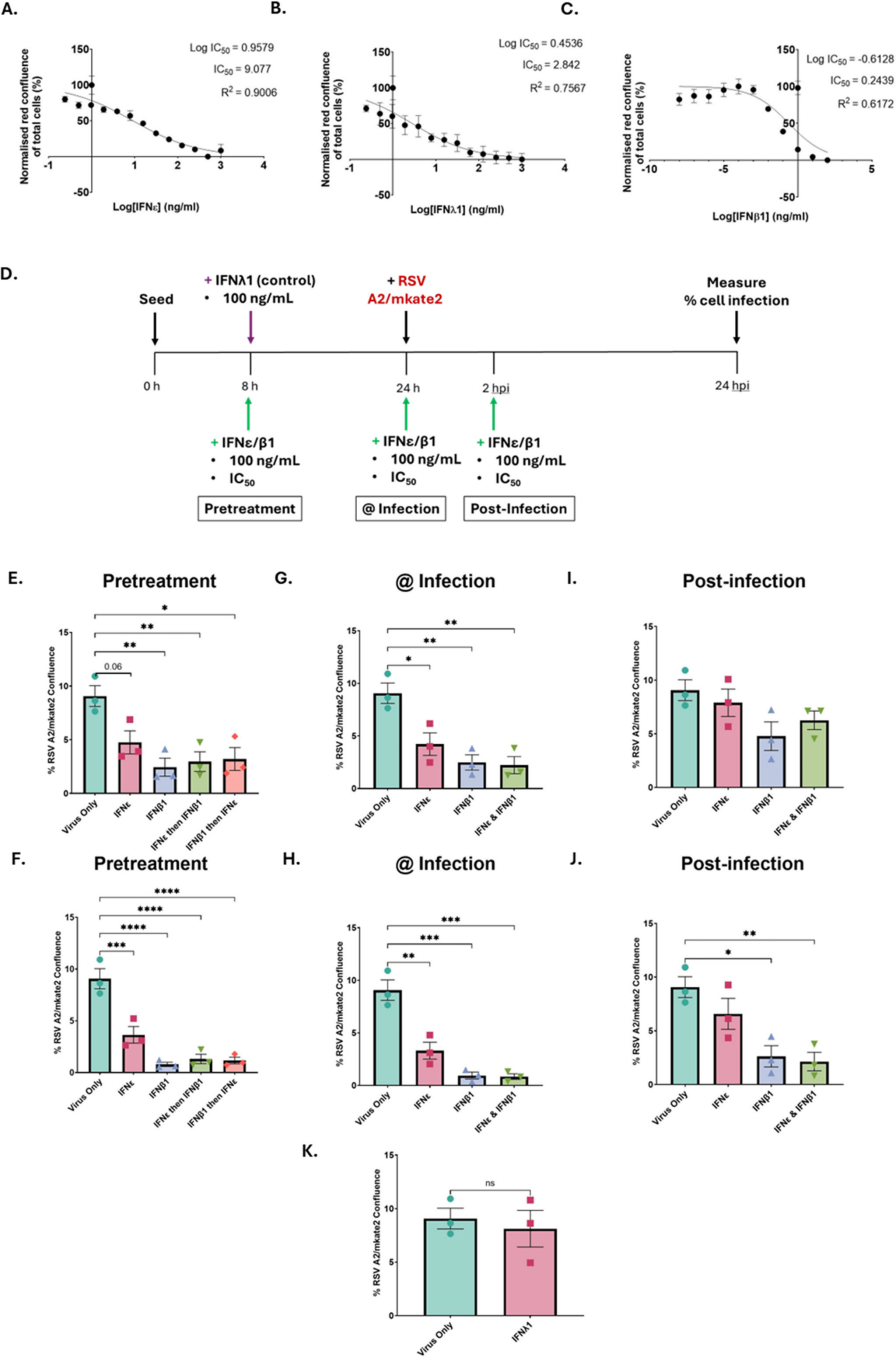
IFNε possesses an increased IC_50_ concentration against RSV compared to IFNβ1. BEAS-2B cells were pre-treated for 16 h with decreasing concentrations of (**A**) rhIFNε, (**B**) rhIFNλ1, and (**C**) rhIFNβ1. Cells were then infected with RSV-A2/mKate2 (MOI = 0.1). At 24 hpi, mean % fluorescence ratio was measured using the brightfield and red fluorescence channels of the Celigo Imaging Cytometer (Nexcelom). Statistical analysis (**A–C**) was conducted by fitting a non-linear dose-response curve for mean of triplicate technical replicates for *n* = 2 (rhIFNε and rhIFNλ1) or *n* = 3 (rhIFNβ1) independent experiments. (**D**) Summary of experimental set-up for pretreatment, treatment at infection, and treatment post-infection experiment. *IFNLR1* Cas9 knockout BEAS-2B cells (*IFNLR1^−/^*^−^ BEAS-2Bs) were either pretreated (**E and F**), treated at infection (**G and H**), or treated 2 h post-infection (**I and J**) with the IC_50_ concentration (**E, G, I**) or 100 ng/mL (**F, H, J**) of rhIFNε and rhIFNβ1 protein. Cells were infected with RSV-A2/mKate2 (MOI = 0.1). (**K**) *IFNLR1^−/^*^−^ BEAS-2B cells were pretreated with 100 ng/mL of rhIFNλ1. (E–J) Data presented as % fluorescence ratio mean of triplicate technical replicates (±SEM) for *n* = 3 independent experiments at 24 hpi. Statistical analysis was conducted using one-way ANOVA followed by Tukey's multiple comparisons test (*P < 0.05, **P < 0.01, ***P < 0.001, ****P < 0.0001). (K) Statistical analysis was conducted using an unpaired two-tailed Student’s *t*-test.

To further facilitate meaningful comparisons of the relative antiviral activity of rhIFNε with rhIFNβ1 and rhIFNλ1, we determined the IC_50_ of these IFNs against RSV A2/mKate2 in BEAS-2B cells. Cells were pre-treated with decreasing concentrations of rhIFNε (Fig. 4A), rhIFNλ1 (Fig. 4B), and rhIFNβ1 (Fig. 4C) and infected with RSV-A2/mKate2 (MOI = 0.1). As anticipated, IFNε possessed the highest best-fit IC_50_ value (9 ng/mL), followed by IFNλ1 (2.8 ng/mL) and IFNβ1 (0.24 ng/mL).

Given the limited understanding of how IFNε exerts its antiviral activity, we aimed to confirm that its presence would not interfere with the activity of other type I IFNs, specifically IFNβ1, in our study. Therefore, to further explore this without the interference from type III IFNs, *IFNLR1*^−/−^ BEAS-2B cells (Fig. 4D) were pre-treated (Fig. 4E and F), treated at infection (Fig. 4G and H), or at 2 h post-infection (Fig. 4I and J) with the IC_50_ concentrations (Fig. 4E, G and I) or 100 ng/mL (Fig. 4F, H and J) of rhIFNε and rhIFNβ1. Cells were infected with RSV-A2/mKate2 (MOI = 0.1). At 24 hpi, IC_50_ concentrations of rhIFNε and rhIFNβ1 resulted in a reduction in viral spread with pre-treatment and with treatment at infection (Fig. 4E and G). However, IC_50_ concentrations of rhIFNε and rhIFNβ1 were not sufficient to significantly diminish RSV spread when treatment commenced 2 hpi (Fig. 4I). When pre-treated with both IFNs sequentially (rhIFNε and then rhIFNβ1 or vice versa) or treated with a combination of the two IFNs at the same time, the antiviral activity of rhIFNβ1 was not altered in the presence of rhIFNε. The greatest reduction in virus spread was seen when rhIFNβ1 was present, regardless of the sequence of addition. Cells pre-treated or treated at infection with 100 ng/mL resulted in robust inhibition of viral spread, with those treated with 100 ng/mL rhIFNβ1 showing the greatest reduction (Fig. 4F and H). Only 100 ng/mL of rhIFNβ1 inhibited viral spread significantly with treatment post-infection (Fig. 4J). rhIFNλ1 pre-treatment (100 ng/mL) in *IFNLR1^−/^*^−^ BEAS-2B cells did not result in a significant reduction in virus spread (Fig. 4K). These results demonstrated that rhIFNε does not interfere with the antiviral activity of rhIFNβ1, despite both binding to the same receptor, at IC_50_ concentrations or 100 ng/mL.

### IFNε induces antiviral activity against Sendai virus but not SARS-CoV-2

**Fig 5:**
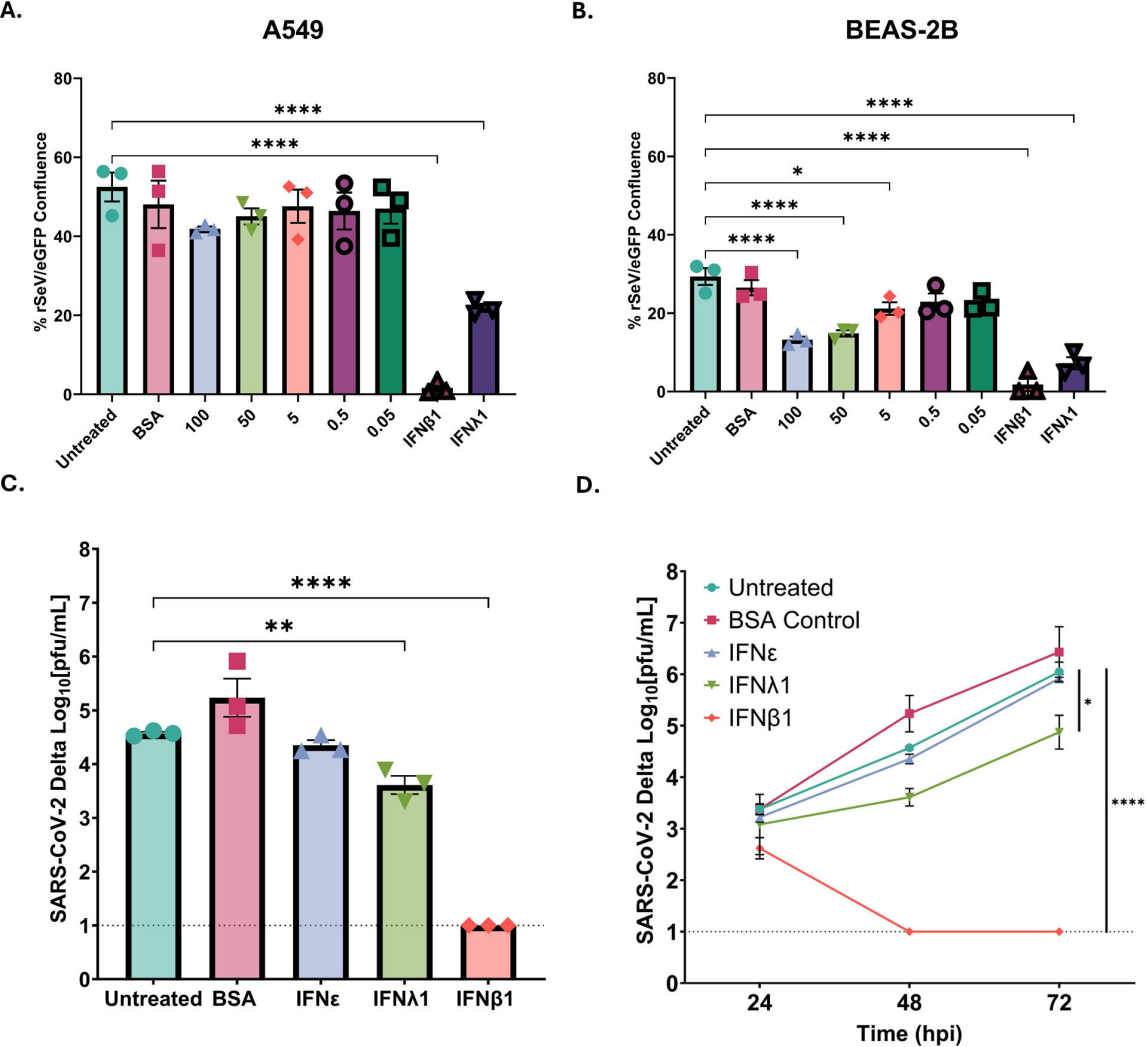
Relative potency of rhIFNε, rhIFNλ1, and rhIFNβ1 pre-treatment against Sendai virus and SARS-CoV-2. (**A**) A549 and (**B**) BEAS-2B cells were pre-treated for 16 h with rhIFNε (100, 50, 5, 0.5, and 0.05 ng/mL), rhIFNβ1, or rhIFNλ1 (100 ng/mL). Cells were infected with rSeV/eGFP (MOI = 0.3). At 24 hpi, mean % fluorescence ratio was measured using the brightfield and green fluorescence channels of the Celigo Imaging Cytometer (Nexcelom). Data presented (A and B) as % fluorescence ratio for mean of triplicate technical replicates (±SEM) for *n* = 3 independent experiments. Statistical analysis (A and B) was conducted using one-way ANOVA followed by Dunnett's multiple comparisons test. (**C and D**) AAT cells were pre-treated for 16 h with rhIFNε, rhIFNλ1, and rhIFNβ1 (100 ng/mL). Cells were then infected with the SARS-CoV-2 Delta variant (MOI = 0.3). Supernatants were collected at 24, 48, and 72 hpi, and plaque assays were performed on VATs. Data presented as the mean of triplicate technical replicates (±SEM) log_10_ pfu/mL at (**C**) 48 hpi and a (**D**) 24–72 hpi time course for *n* = 3 independent experiments. Statistical analysis (**C**) was conducted using one-way ANOVA followed by Dunnett's multiple comparisons test. (**D**) Statistical analysis was conducted using one-way ANOVA of AUC (*P < 0.05, **P < 0.01, ***P < 0.001, ****P < 0.0001).

To assess if an antiviral effect was evident beyond EMCV and RSV, A549 (Fig. 5A) and BEAS-2B (Fig. 5B) cells were pre-treated with a dose range of rhIFNε (100, 50, 5, 0.5, and 0.05 ng/mL) and infected with rSeV/eGFP (MOI = 0.3). Despite the observed trend toward reduction, A549 cells did not exhibit a significant decrease (Fig. 5A). At 24 hpi, a similar dose-dependent antiviral activity of rhIFNε was evident against rSeV/eGFP, as previously described for RSV A2/mKate2 in BEAS-2B cells (Fig. 5B). The antiviral effect of rhIFNε pre-treatment (2.2-fold reduction) was like that of rhIFNλ1 (4-fold reduction) but less impressive compared to rhIFNβ1 (16.3-fold reduction) (Fig. 5B). In contrast, AAT cells pretreated with rhIFNε, rhIFNβ1, or rhIFNλ1 (100 ng/mL) and infected with the SARS-CoV-2 Delta variant (MOI = 0.3) had no significant reductions in viral replication with IFNε pre-treatment, a slight but significant reduction in virus replication with rhIFNλ1 pre-treatment, and a complete abrogation of virus replication following rhIFNβ1 pre-treatment at 48 hpi (Fig. 5C) and over a time course of infection (Fig. 5D). These results suggest that rhIFNε possesses antiviral activity against some RNA viruses, but not the SARS-CoV-2 Delta variant, at least at the dose tested, and its potency is reduced compared to rhIFNβ1.

### JAK-STAT inhibitor alters IFNε-mediated reduction of viral replication

**Fig 6:**
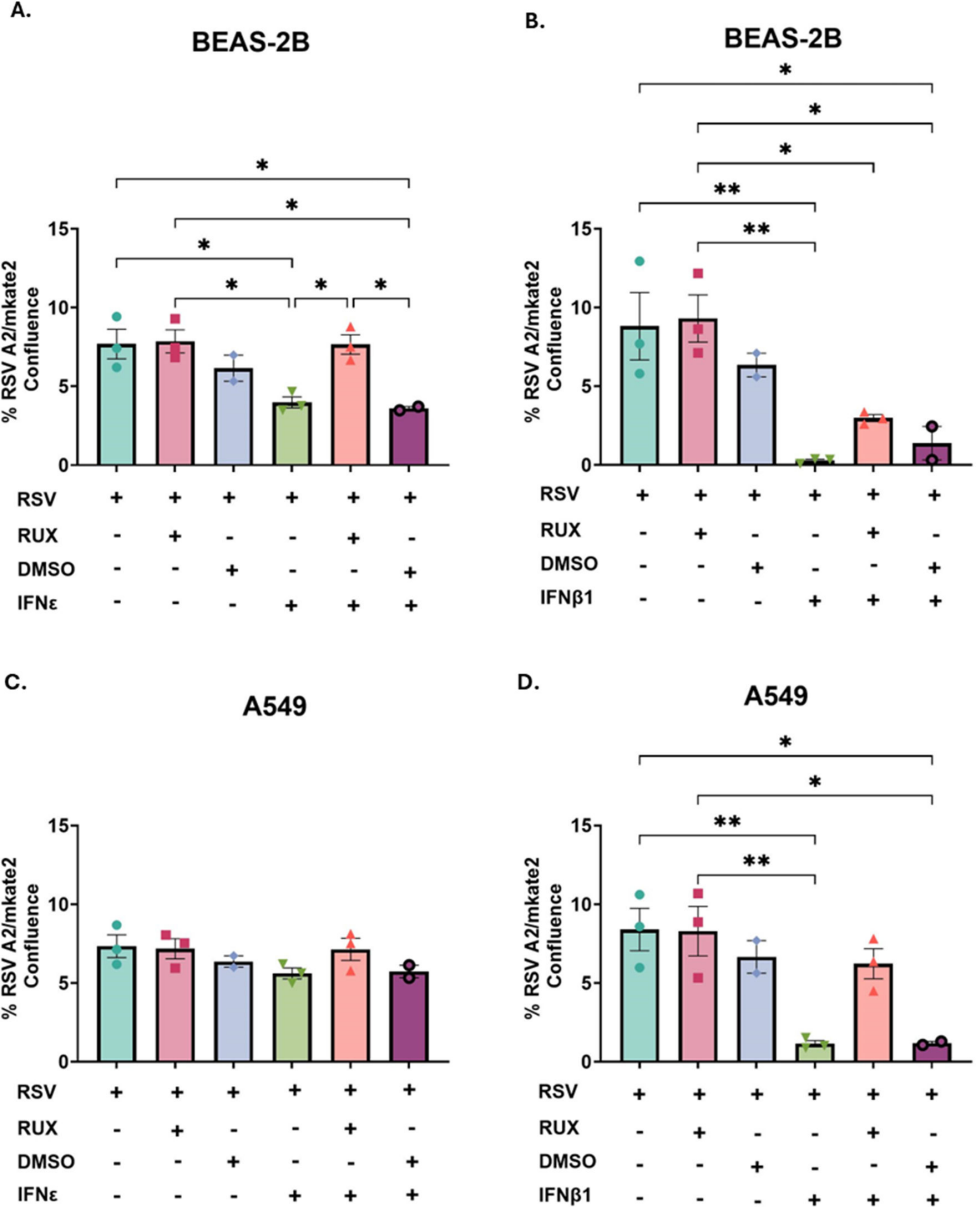
JAK-STAT inhibitor alters IFNε-mediated reduction of viral replication. BEAS-2B (A and B) and A549 (C and D) cells were pre-treated with Ruxolitinib (2.5 µM) (INCB018424, Selleck Chemicals) or DMSO (2.5 µM) for 2 h before 16 h rhIFNε (**A and C**), and rhIFNβ1 (**B and D**), pre-treatment (100 ng/mL). Cells were then infected with RSV (RSV-A2/mKate2) (MOI = 0.1). At 24 hpi, BEAS-2B (**A and B**) and A549 (**C and D**) cells mean % fluorescence ratio was measured using the brightfield and red fluorescence channels of the Celigo Imaging Cytometer (Nexcelom). Data presented as % fluorescence ratio for mean of triplicate technical replicates (±SEM) for *n* = 3 independent experiments for RSV, RSV + RUX, RSV + IFNε, and RSV + RUX + IFNε. Data presented as % fluorescence ratio of triplicate technical replicates for (±SEM) *n* = 2 independent experiments for RSV + DMSO and RSV + DMSO + IFNε. Statistical analysis was conducted using one-way ANOVA followed by Tukey's multiple comparisons test (*P < 0.05, **P < 0.01).

To confirm that IFNε was signaling via the classical type I IFN signal induction pathway (IFNAR-JAK-STAT) in airway epithelial cells, BEAS-2B (Fig. 6A and B) and A549 (Fig. 6C and D) cells were pre-treated with the JAK inhibitor Ruxolitinib for 2 h prior to rhIFNε (Fig. 6A and C) and rhIFNβ1 (Fig. 6B and D) pre-treatment (100 ng/mL). Ruxolitinib was dissolved in DMSO, which was used as a negative control. Pre-treatment with Ruxolitinib eliminated the reduction in viral replication with rhIFNε pretreatment in BEAS-2B cells (Fig. 6A). A similar trend was seen for rhIFNβ1-pre-treated BEAS-2Bs (Fig. 6B) although the concentration of Ruxolitinib used was not sufficient to completely block rhIFNβ1 signaling at 100 ng/mL. A549 cells did not produce a significant reduction in viral replication with rhIFNε treatment (Fig. 6C). However, pre-treatment of A549 cells with 100 ng/mL of rhIFNβ1 did result in a significant reduction in viral replication (Fig. 6D). These results confirm the increased potency of rhIFNβ1 compared to rhIFNε at 100 ng/mL but that, like rhIFNβ1, rhIFNε utilizes the JAK-STAT pathway to exert its antiviral effect against RSV.

### Differential ISG expression kinetics following rhIFNε, rhIFNλ1, and rhIFNβ1 treatment

**Fig 7:**
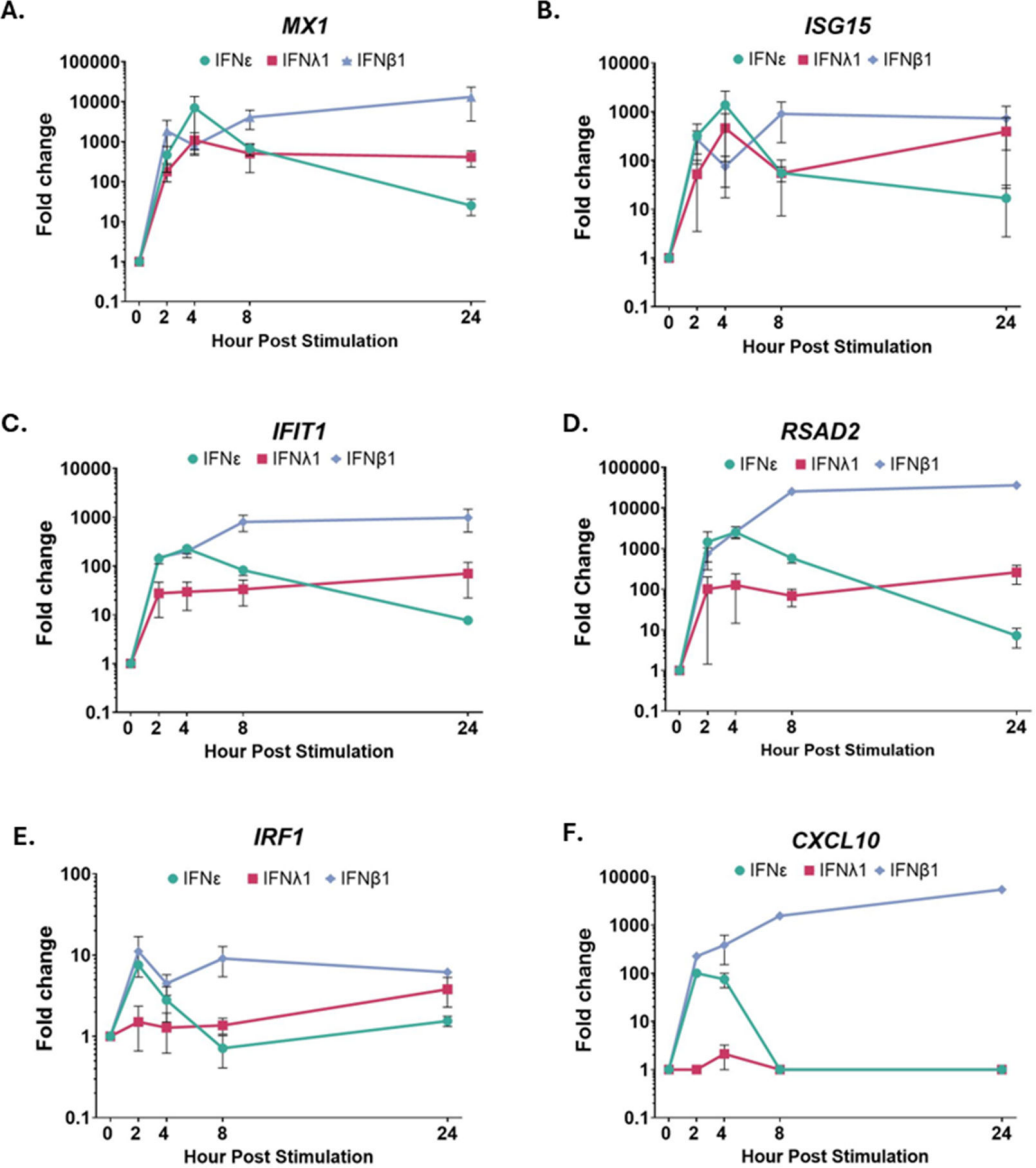
Differential induction of antiviral and pro-inflammatory ISGs by IFNε. BEAS-2B cells were treated with rhIFNε, rhIFNλ1, or rhIFNβ1 (100 ng/mL), and total RNA was extracted immediately from untreated (*t* = 0) and at indicated time points post-stimulation. RNA was subjected to RT-qPCR analysis of *IFIT1*, *ISG15*, *MX1, RSAD2, IRF1,* and *CXCL10* expression (ROCHE, Lightcycler 480). Graphs show relative ratio values for antiviral ISGs (**A**) *MX1*, (**B**) *ISG15*, (**C**) *IFIT1,* (**D**) *RSAD2* and pro-inflammatory ISGs (**E**) *IRF1* and (**F**) CXCL10, normalized using *YWHAZ*, *IPO8,* and *TBP* as housekeeping genes. Data presented (**A–D**) are mean of duplicate technical replicates (±SEM) fold change from mock for *n* = 3 independent experiments, and data presented (**E and F**) are mean of duplicate technical replicates (±SEM) fold change from mock for *n* = 2 independent experiments.

In our hands, IFNε was capable of inhibiting RSV replication only at early time points post-infection suggesting that the expression of downstream antiviral restriction factors may be transient. To assess if any differences were evident in the expression kinetics of downstream antiviral restriction factors with rhIFNε treatment compared to rhIFNβ1 and rhIFNλ1, RT-qPCR was conducted on RNA extracted from BEAS-2B cells treated with rhIFNε, rhIFNλ1, and rhIFNβ1 (100 ng/mL) (Fig. 7A through F). rhIFNε induced well-known antiviral proteins, such as *MX1* (Fig. 7A), *ISG15* (Fig. 7B), *IFIT1* (Fig. 7C), and *RSAD2* (Fig. 7D). Similarly, IFNε also induced type I IFN-associated pro-inflammatory genes, such as *IRF1* (Fig. 7E) and *CXCL10* (Fig. 7F), which were previously reported to be induced only at low levels and transiently by rhIFNλ1 or other type III IFNs (41). rhIFNε possessed unique ISG expression kinetics, with expression peaking early (4 h post-treatment), in comparison to rhIFNβ1, which induced a more potent and enduring response, and rhIFNλ1, which induced a slower yet incrementally increasing response through 24 h post-treatment. These results demonstrate that rhIFNε can induce a typical pro-inflammatory type I IFN antiviral gene signature in airway epithelial cells but that temporal expression of downstream ISGs is transient compared to rhIFNβ1 and rhIFNλ1 treatment.

## DISCUSSION

Conflicting data exist surrounding the role of IFN responses during RSV infection, particularly among those most vulnerable to severe RSV-related disease, infants aged 6 weeks to 6 months of life. Most of what is known describes the “classical” type I (IFNβ1 and IFNα) and type III (IFNλ1-3) IFNs (42, 43). However, little is understood about the “non-classical” IFN subtypes, such as IFNε. Previously, we exploited a unique cohort of primary pediatric airway epithelial cell samples derived from the same healthy infants at birth and 1 year, to explore the development of innate immune responses to RSV infection in the first year of life (23). We present the differential expression of *IFNE* in response to RSV infection in 1-year-old infants compared to uninfected controls, as well as with chronological age, by comparing newborns and 1-year-old infants, both at baseline and following RSV infection in WD-PNEC cultures. This timespan coincides with those infants with high susceptibility rates to severe RSV-related disease (newborn) and those with lower frequencies of severe infection (1 year). Previous studies have reported endogenous and infection-induced *IFNE* expression in both the upper and lower respiratory tracts in humans and other mammals, with variations in expression linked to age and disease severity (26, 31, 32, 44, 45). IFNε is conserved across various mammalian species (26, 46–49), but is pseudogenized in pangolins, which are susceptible to a broad range of human viral infections, including RSV and SARS-CoV-2. While this suggests *IFNE* may not be essential for antiviral defense in all species, its absence in highly susceptible hosts may imply a role in immunity. Therefore, in species where *IFNE* remains functional, its conserved mucosal expression pattern points to a potentially important evolutionary role in mucosal immunity, distinct from that of classical IFNs (50–52). For example, *IFNE* is expressed constitutively at higher levels in bat derived nasal and bronchial air–liquid interface cultures compared to equivalent human cultures, where it is associated with the basal expression of multiple ISGs (53). These findings demonstrate that in species such as bats, known for their heightened resistance to highly human-pathogenic viruses, constitutive ISG expression, driven by constitutively active factors such as *IFNE*, contribute to a baseline protective antiviral state. Collectively, these data suggest that IFNε may play a protective role in the respiratory epithelium during human early-life viral infection and elevated levels in older infants may be an indication of an improved ability to curtail and/or resolve infection.

As epithelial cells are the primary target of RSV infection *in vivo*, we assessed the expression of *IFNE* in immortalized epithelial cell lines commonly used to model RSV infection. *IFNE* was basally expressed at low levels in all cell lines tested. Recently, Martínez-Espinoza et al. described increased *IFNE* expression during infection with RSV and human metapneumovirus (hMPV), with peak *IFNE* expression at 48 hpi in A549 cells for both viruses (44). We did not see this increase in *IFNE* expression with RSV infection. This discrepancy may be due to various factors, including differences in cell culturing conditions, viral strains used, assays performed, and other experimental variables between authors. Our observations are consistent with its reported unstimulated basal expression within the epithelium of the FRT and male reproductive tract in humans and mice (24, 25, 27, 54, 55). However, we did observe an increase in *IFNE* expression within our RSV-infected WD-PNEC cultures, mirroring the increase Martinez-Espinoza et al. reported within human bronchial airway epithelial cells 3–5 days post-RSV-infection (44). Notably, Martínez-Espinoza et al. suggested that RSV and hMPV induced *IFNE* expression in A549 cells via RIG-I, with contributions from MDA5 (44). This suggests that viral RNA plays a role in the induction of *IFNE* during active infection. The basal expression of *IFNE* is conserved across multiple mucosal sites, concentrated primarily in the epithelial cells lining these surfaces. Consequently, it may be regulated by distinct stimuli in a cell/tissue-specific manner, possibly by distinct cell populations in these sites, which fluctuate with tissue-specific stimuli. However, no studies have been published regarding IFNε’s role in human respiratory epithelium on a single-cell level, and further work needs to be conducted regarding *IFNE* induction and regulation in more physiologically relevant primary human airway epithelial models.

As a result of *IFNE*’s basal epithelial expression, further investigation was prompted regarding its possible contribution to localized antiviral activity. We showed that rhIFNε provided antiviral protection against EMCV infection in BEAS-2B and A549 cells, consistent with similar antiviral effects observed in cervical WISH cells (28). Martínez-Espinoza et al. demonstrated the antiviral activity of rhIFNε against RSV and hMPV in A549 cells (44), which were shown to be highly permissive to RSV infection (56). Therefore, we chose to assess the antiviral properties of rhIFNε in multiple respiratory cell lines. Pre-treatment with rhIFNε led to a significant, dose-dependent reduction in viral replication in both BEAS-2B and HEp-2 cell lines. Notably, BEAS-2B cells are known to mount robust antiviral responses, expressing high levels of transcription factors and ISGs upon RSV infection or IFN stimulation (56). This inherent strong responsiveness likely explains their pronounced reaction to rhIFNε treatment in our experiments. We did not observe a significant reduction in virus fluorescence in A549 and AAT cells when treated with 100 ng/mL of rhIFNε, consistent with Martínez-Espinoza et al., who found that at least 250 ng/mL was needed for a significant effect on RSV fluorescence or titres in A549 cells (44). In addition, prototypic lab-adapted strains, such as RSV A2/mKate2, have been reported to result in greater cytopathogenesis and induce greater concentrations of proinflammatory cytokines in the respiratory epithelium (36). Therefore, we also chose to assess the antiviral activity of rhIFNε against a low passage clinical isolate of RSV, which more accurately reflects RSV infection *in vivo*. We found that rhIFNε pretreatment of BEAS-2B cells was antiviral against the clinical isolate RSV BT2a, with a significant reduction in infectious viral release only at 24 hpi. However, without being replenished, this antiviral effect was quickly lost as the infection progressed. BEAS-2B cells were selected as the representative cell line due to their significant reduction in RSV A2/mKate2 infection following 100 ng/mL rhIFNε pretreatment and their non-cancerous immortalized state. Our data emphasize the importance of cell line selection in studying viral IFN sensitivity, especially for lesser-understood IFN subtypes, and suggest that while IFNε may not be the primary defender against viral infection in epithelial cells, it supports its potential role as a basal antiviral mucosal defense cytokine.

The relative antiviral activities of each IFN were reflected in their IC_50_ values. Our data indicated that rhIFNε was ~3-fold less potent than rhIFNλ1 and ~38-fold less potent than rhIFNβ1 similar to those described for recombinant mouse IFNε (rmIFNε) against EMCV in WISH cells (28). Interestingly, the IC_50_ concentration of rhIFNε and 100 ng/mL was sufficient to reduce viral replication under pretreatment and treatment-at-infection conditions, but not with treatment 2 h post RSV infection in BEAS-2B cells. However, its inability to provide protection against infection when added post-RSV infection most likely reflects its reduced antiviral potency and RSV’s recognized ability to efficiently modulate IFN responses (57). This is consistent with data showing a reduction in Zika virus (ZIKV) replication in HRT8 cells with rmIFNε pretreatment, but not post-infection (54). As expected, RSV was also able to antagonize rhIFNβ1’s antiviral activity when added post-infection at its IC_50_ concentration, as IFNε and IFNβ1 share the same signaling pathway. However, when added 2 h post-RSV infection at 100 ng/mL, rhIFNβ1 was able to reduce viral replication significantly, most likely reflecting rhIFNβ1’s greater potency. As anticipated, the antiviral activity of rhIFNβ1 consistently superseded rhIFNε when added before, after, or simultaneously with rhIFNε. Like IFNβ1, IFNε possesses a greater affinity for the IFNAR1 chain compared to the IFNAR2 chain, with IFNAR1 acting as the high-affinity binding chain, a pattern that is atypical among other type I IFN subtypes (28). However, IFNε binds to IFNAR1 at a much lower affinity than IFNβ1 (100- to 1,000-fold), which may partly explain the increased antiviral capacity of IFNβ1 in this context (28).

Infection with the related virus rSeV/eGFP confirmed that rhIFNε’s antiviral effect was not restricted to RSV, with the greatest reduction in rSeV/eGFP fluorescence also observed within BEAS-2B cells. However, we did not observe an antiviral effect against the SARS-CoV-2 Delta variant in AAT cells at the high dose tested (100 ng/mL), a variant that was previously described to have increased sensitivity to IFNs (58–61). To our knowledge, this is the first report demonstrating SARS-CoV-2’s insensitivity to rhIFNε (62). Although our results suggest that rhIFNε was not effective in altering SARS-CoV-2 infection, it is important to note that we used a modified immortalized cell line highly susceptible to SARS-CoV-2 infection. Further studies in a more physiologically relevant nasal epithelial model are required, given evidence that IFNε may influence age-related responses to SARS-CoV-2 (32, 33). It is possible that during SARS-CoV-2 infection, IFNε possesses functions beyond direct antiviral activity, as suggested by its wider immunomodulatory functions (63). Overall, our data demonstrate that respiratory viruses possess differential sensitivities to rhIFNε restriction and emphasize the importance of assessing the antiviral capacity against multiple virus families.

Our data using a JAK-STAT signaling inhibitor confirmed that IFNε signals through this pathway to exert its antiviral activity against RSV. These data provided the rationale to explore the kinetics of the induction of downstream ISGs with rhIFNε treatment. Recently, IFNε was shown to induce common ISGs upregulated during RSV infection in A549 cells (44). Our data showed that rhIFNε treatment induced a unique ISG expression pattern with expression of four antiviral ISGs associated with RSV infection (*MX1*, *ISG15*, *IFIT1,* and *RSAD2*) peaking at 4 h post-stimulation. IFNε’s transient induction pattern was consistent with that observed against ZIKV infection in human FRT cell lines (54), and this rapid transient ISG induction may be reflective of its short-term reduction in viral titer at 24 hpi in BEAS-2B cells, but not at later time points. Furthermore, rhIFNε, like rhIFNβ1, induced the expression of the transcription factor *IRF1* and the chemokine *CXCL10*. rhIFNλ1 treatment did not induce *IRF1* or *CXCL10* expression to the same magnitude compared to rhIFNε and rhIFNβ1. Rapidly increased *IRF1* and *CXCL10* expression is considered type I IFN-associated (41). However, type III IFNs were shown to induce *IRF1* and *CXCL10* expression at lower levels (41). Together, our results demonstrated that rhIFNε can induce a strong classical type I antiviral gene signature in airway epithelial cells, but its potency and expression kinetics are distinct compared to rhIFNβ1 and rhIFNλ1.

IFNε’s unique low-level basal expression, its reduced potency, and transient ISG expression kinetics may strike a balance between the beneficial effects of type I IFN and minimizing the tissue toxicity associated with its presence (64). The question remains whether the basal expression of IFNε results in a continuously primed airway epithelium, creating a basal barrier to infection. If so, IFNε or the ISGs it induces could be leveraged as potential biomarkers to identify those children at increased risk of severe disease in which no high-risk factors have been identified (6 weeks and 6 months). Could the unique properties of IFNε be further harnessed as an antiviral to non-destructively boost protective responses to infection? Further work is required to explore IFNε’s ability to boost or improve innate immune responses within primary airway epithelial cell cultures.

### Conclusion

IFNε is a highly conserved type I IFN expressed at multiple mucosal surfaces and is recognized for its antimicrobial abilities in the FRT. We demonstrated differential expression of *IFNE* with age and RSV infection in human nasal epithelial cells and confirmed its antiviral activity against a clinical isolate of RSV. Although less potent than rhIFNβ1 or rhIFNλ1, rhIFNε’s ISG expression kinetics at 100 ng/mL and mild inflammatory profile suggest it may play a role in mitigating RSV infection in airway epithelium. Reduced IFNε expression in newborns may partially explain their heightened vulnerability to RSV, presenting an opportunity for prophylactic interventions. Further studies are needed to explore IFNε’s potential as an antiviral therapy or prognostic tool for early-life respiratory viral infections.

## Data Availability

RNA-seq data have been deposited in the NCBI GEO database under accession number GSE231666.
